# Pain Assessment Tools for Infants, Children, and Adolescents With Cancer: Protocol for a Scoping Review

**DOI:** 10.2196/66614

**Published:** 2025-04-28

**Authors:** Mika Hirata, Noyuri Yamaji, Shotaro Iwamoto, Ayaka Hasegawa, Mitsuru Miyachi, Takashi Yamaguchi, Daisuke Hasegawa, Erika Ota, Nobuyuki Yotani, Japanese Society for Palliative Medicine

**Affiliations:** 1 Department of Child Health Nursing Graduate School of Health Care and Nursing Juntendo University Chiba Japan; 2 Institute of Clinical Epidemiology Showa Medical University Tokyo Japan; 3 Global Health Nursing Graduate School of Nursing St. Luke's International University Tokyo Japan; 4 Department of Family Nursing Graduate School of Medicine The University of Tokyo Tokyo Japan; 5 Mie Kids ＆ Family Home Care Clinic Mie Japan; 6 Pharmaceutical Department National Center For Child Health and Development Tokyo Japan; 7 Department of Pediatrics Graduate School of Medical Science Kyoto Prefectural University of Medicine Kyoto Japan; 8 Department of Palliative Care Konan Medical Center Hyogo Japan; 9 Department of Pediatrics St. Luke’s International Hospital Tokyo Japan; 10 Division of Palliative Medicine National Center for Child Health and Development Tokyo Japan

**Keywords:** pediatric, infants, children, adolescents, pediatric cancer, developmental stage, pain assessment tool, scoping review

## Abstract

**Background:**

Pain management in children with cancer may be inadequate due to poor pain assessment, and evaluation using suitable tools is necessary. Despite the availability of many pain assessment scales, few studies have summarized the existing assessment tools, making it challenging to select a suitable scale.

**Objective:**

This scoping review aims to map existing pain assessment tools for children with cancer and provide a comprehensive overview of pediatric cancer-related pain screening and assessment tools.

**Methods:**

The scoping review will be conducted according to the guidelines by the Joanna Briggs Institute and reported following the PRISMA-ScR (Preferred Reporting Items for Systematic Reviews and Meta-Analyses Extension for Scoping Reviews) framework. Electronic databases, including PubMed, CINAHL, CENTRAL, ICHUSHI (Japan Medical Abstracts Society), and Embase, will be searched to identify eligible studies, without date or language restrictions. We defined the eligibility criteria based on the PCC (Population, Concept, and Context) format. Studies that focused on assessment tools for evaluating pain in children (aged 0-18 years) with cancer in a hospital or at home will be included. Although there are no restrictions on study design, protocols and conference abstracts will be excluded. Two or more reviewers will select studies by reviewing the full text of relevant articles identified by titles and abstracts, and disagreements will be resolved through discussion. Two or more reviewers will extract predefined data items, including characteristics of included studies (eg, author name, title of publication, year of publication, purpose of study, study setting, study population, outline of the assessment tool, study design, and findings) and the characteristics of assessment tools (eg, types of tools, target population, assessor, validity, instructions, precautions, and advantages and disadvantages of the tools). Pain assessment tools will be summarized in tabular format and described in a narrative synthesis.

**Results:**

Through electronic database searches on November 20, 2023, we identified 3748 articles. This review will provide a comprehensive overview of pain assessment tools. The final report is planned for submission to a peer-reviewed journal in 2025.

**Conclusions:**

This scoping review is the first comprehensive effort to map existing tools on pediatric cancer-related pain assessment tools for infants, children, and adolescents aged <18 years, according to developmental stages. Based on the findings of this study, we will discuss future clinical and research implications for pain assessment and management in children with cancer. The findings are expected to enhance pain management practices in children with cancer and inform health care providers, policy makers, and other stakeholders.

**International Registered Report Identifier (IRRID):**

DERR1-10.2196/66614

## Introduction

Annually, an estimated 400,000 children and adolescents aged 0-19 years are diagnosed with all types of cancer [1,2]. Due to advances in medical care, >80% of these children will become long-term survivors in high-income countries, while <30% will be cured in low- and middle-income countries [1,3]. In Japan, 2000 diagnoses of childhood cancer are issued annually, and the 5-year survival rate is reported to be 80% [4].

Palliative care for children refers to the active total care of the child’s body, mind, and spirit, and involves providing support to the family. It begins when the illness is diagnosed and continues regardless of whether a child receives treatment directed at the disease [5]. Children with cancer experience pain resulting from the disease itself, medical treatments (eg, chemotherapy and radiation therapy), and procedures (eg, lumbar punctures and bone marrow punctures), from diagnosis to end of life or post treatment [6]. Pain is one of the most distressing experiences for children during treatment [7]. Studies have shown that 39% of children undergoing cancer treatment and 58% of children who are terminally ill experience severe pain-related distress [8]. Schulte et al [9] suggest that ineffective pain management during treatment, combined with lifestyle factors, late effects, and psychological stress, may lead to long-term chronic pain. Moreover, the child’s experience of pain affects family members and is one of the factors that increase family emotional distress during treatment [10]. This distress, in turn, is transmitted to the child, creating a vicious cycle that exacerbates the patient’s pain [11].

Underlying the inadequate pediatric cancer pain management is the lack of proper pain assessment, which is key in initiating pharmacologic or nonpharmacologic therapy to assess and modify the effectiveness of treatment and care. Self-report is the gold standard for pain assessment [12], and a 2015 report by Wolfe-Christensen et al [13] found that 99% of children older than 13 years and 96% of children aged 7-12 years can reliably self-report pain. Conversely, children express pain differently depending on their developmental stage and cognitive abilities, requiring the use of differing tools for pain assessment. Therefore, observation of developmental stage-specific expressions, selection of suitable pain assessment tools, and appropriate interventions are necessary. In addition, studies suggest that parents often detect their child’s pain more quickly than health care providers do [14]. In a 2019 report, Madden et al [15] indicated that both children and caregivers rated pain and lack of appetite as the most distressing symptoms, with a strong correlation between children’s and parents’ reports of pain (*r*_s_>0.8). Therefore, parental involvement must also be considered in the assessment of pain in children. Although many tools for pain assessment exist [16], few studies have mapped availability based on children’s developmental stages. This study aims to map existing studies on the assessment of cancer-related pain in pediatric patients. Moreover, it summarizes the characteristics of children’s expression of cancer pain across developmental stages, as well as tools, instruments, and indicators used for pain assessment and their associated outcomes. This study is also expected to summarize parental involvement and outcomes of cancer-related pain assessment. Based on the findings, the need for future research on pain management in pediatric cancer will be discussed. Thus, our primary goal is to synthesize existing literature on cancer-related pain assessment tools for infants, children, and adolescents aged <18 years, ultimately contributing to more accurate pain assessments essential for effective pain management.

## Methods

### Study Design

We will conduct a scoping review following the Joanna Briggs Institute (JBI) Manual for Evidence Synthesis [17]. Research team members discussed and revised the drafted protocol. The final report will be presented following the PRISMA-ScR (Preferred Reporting Items for Systematic Reviews and Meta-Analyses Extension for Scoping Reviews) [18]. This study will address the question, “What pain assessment tools are available to measure pediatric cancer-related pain according to the child’s developmental stages?”

### Eligibility Criteria

The eligibility criteria will be decided based on the PCC (Population, Concept, and Context) format and type of study [17].

#### Population

We will include studies that have targeted infants, children, and adolescents aged between 0 and 18 years [19] with any type (eg, leukemia, tumor, or sarcoma) and any phase (eg, prediagnosis, during treatment, and posttreatment) of cancer. If the studies have focused on a mixed population, including adults and those with other diseases, we will include the studies in which over 80% of the population meets our eligibility criteria.

#### Concept

We will include studies that have investigated, described, and validated assessment tools, including scales, instruments, questionnaires, charts, worksheets, and measurements, to assess cancer-related pain. Cancer-related pain was defined as pain caused by the cancer itself and cancer treatments (eg, chemotherapy, stem cell transplantation, immunotherapy, hormone therapy, or radiation). We will include studies that have investigated comprehensive symptom assessment tools if they also include pain assessment as an item. However, we will exclude studies if these tools assess other concepts, such as quality of life. Self-assessment by children with cancer and proxy pain assessment by parents, caregivers, and health care professionals will also be included. While cancer pain is often chronic and multifaceted, procedural and postoperative pain is typically acute and directly related to specific interventions and time limitations [20,21]. Thus, to focus more on cancer-related pain, studies that have focused on procedural or postoperative pain will be excluded. Studies that use measurement tools without providing details about them, such as those evaluating the effectiveness of an intervention, will also be excluded.

#### Context

We will include studies conducted in any health care (eg, hospitals, hospices, and clinics) and home settings to assess pain in children with cancer.

#### Types of Studies

We will include both quantitative and qualitative studies, as well as systematic reviews published in English and Japanese. In particular, we will include the following studies: experimental and quasi-experimental (eg, randomized controlled trials, nonrandomized controlled trials, before and after studies), observational (eg, prospective and retrospective cohort, case-control, and cross-sectional studies), descriptive observational (eg, case series, individual case reports, and descriptive cross-sectional), and exploratory (eg, in-depth interviews and focus group) studies. To map detailed information regarding pain assessment tools, we will exclude protocols and abstracts. Moreover, to ensure the quality of the studies, we will exclude gray literature.

### Search Strategies and Information Sources

We will search electronic bibliographic databases, including PubMed, CINAHL, CENTRAL, Japan Medical Abstracts Society (ICHUSHI), and Embase to identify relevant studies using text words and index terms at a facility. Search strategies will be developed with a health librarian for each database. We will use keywords based on the PCC format, including “children,” “cancer,” “assessment tool,” and “pain.” Our search strategy for PubMed is shown in Multimedia Appendix 1. Moreover, we will search the identified relevant studies by checking the reference lists of included studies and textbooks. We will not set any limits on the search period and language.

### Study Selection

**Figure 1:**
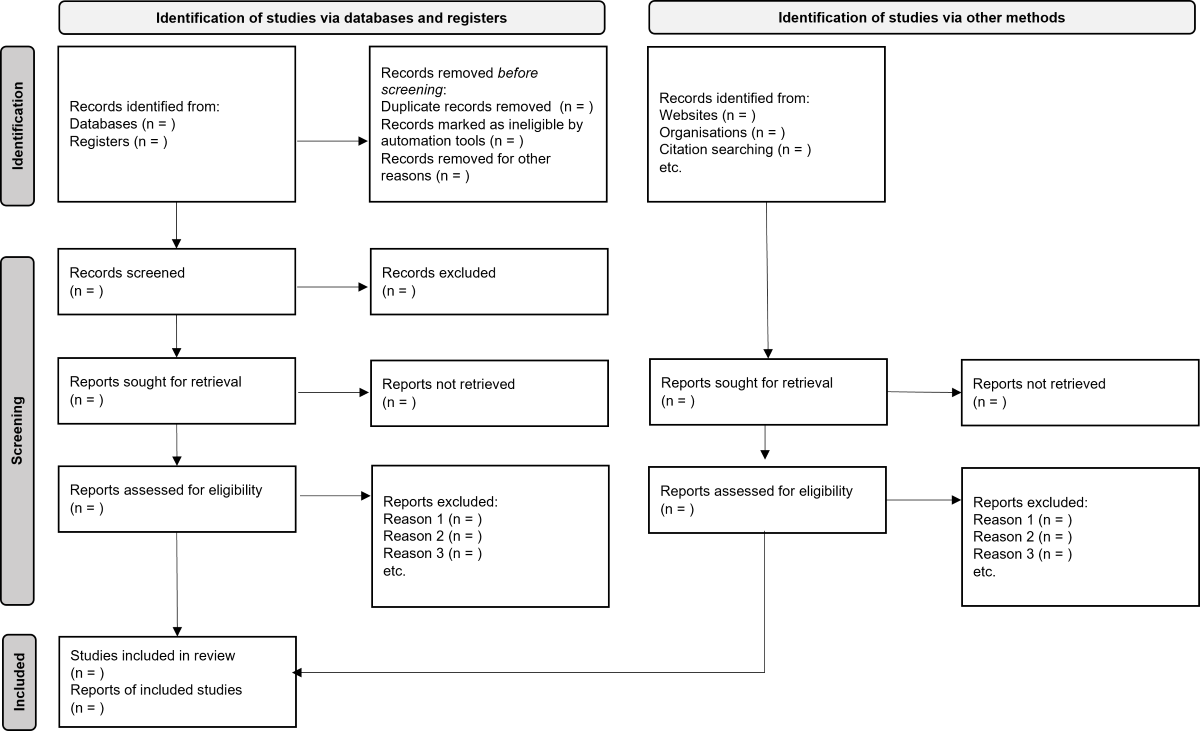
PRISMA (Preferred Reporting Items for Systematic Reviews and Meta-Analyses) flow diagram.

In this study, we will organize a research team. We will screen using Rayyan [22] and report the relevant studies following the PRISMA flow diagram (Figure 1). All search results will be retrieved and imported to a reference management software (EndNote, Clarivate) and duplicate entries will be removed by checking them individually. First, two or more reviewers will independently read the title and abstract and assess the studies that meet our eligibility criteria. Second, two or more reviewers will independently read the full text and assess the eligible studies. Disagreements will be resolved by discussion, and a third author will be included if needed.

### Data Extraction

The research team collaboratively developed a data chart in Microsoft Excel to extract and record data based on the research question. Initially, we intend to use this format for one or two studies and refine it accordingly, if necessary. Two or more reviewers will extract the relevant data from the included studies.

### Data Items

We will collect data on the characteristics of the included studies and assessment tools.

The following characteristics will be assessed: author name, title of publication, year of publication, purpose of study, study setting, study population, outline of the assessment tool, study design, and findings (Table S1 in Multimedia Appendix 2).

The characteristics of assessment tools will include types of tools (eg, scale, instrument, or measurement), target population, assessor (eg, self, parents, caregivers, health care professionals), validity (including for Japanese children with cancer), instructions, precautions, and advantages and disadvantages of the tools (Table S2 in Multimedia Appendix 2).

### Critical Appraisal of Individual Sources of Evidence

We have no plans to assess the risk of bias in individual studies, as the review aims to map the current evidence rather than synthesize study findings.

### Synthesis of Results

The characteristics of the included studies and of assessment tools used to assess cancer-related pain in pediatric patients will be summarized in a tabular format and narratively described. In this synthesis, we will stratify self- and peer-administered assessment tools and present detailed information (eg, appropriate age range, types of pain, and instructions). Subsequently, we will discuss the selection of assessment tools for more accurate pain assessment and the identified research gaps.

### Ethics and Dissemination

Formal ethical approval is not required, as primary data will not be collected in this study. The findings will be disseminated through a presentation at a conference and publication in a peer-reviewed journal.

## Results

We conducted a database search on November 20, 2023, and identified 3748 articles related to pain assessment tools for children with cancer. This study will map these tools based on the existing evidence, providing a comprehensive overview of pediatric cancer-related pain screening and assessment tools. The final report is planned for submission to a peer-reviewed journal in 2025.

## Discussion

To the best of our knowledge, this is the first comprehensive scoping review aimed at investigating pediatric cancer-related pain screening and assessment tools and at providing detailed information, such as target population, assessors, instructions, and validation. Although many pain assessment tools exist [16], few studies have mapped the available pain assessment tools and their instructions according to pediatric developmental stages.

The findings from this comprehensive scoping review would inform pain management practices in pediatric oncology. Health care professionals can be better equipped to select the most appropriate assessment tools by mapping available tools considering developmental stages, assessors, and instructions for various tools. This tailored approach will lead to more accurate pain assessments, which is crucial for effective pain management. Moreover, a clearer understanding of available tools and their applications could enhance communication among health care professionals, children, and families regarding pain assessment. Respecting children’s voices will foster more child- and family-centered care approaches. Ultimately, this may improve pain management outcomes and the quality of life for children with cancer-related pain. These findings may also serve as a valuable resource for health care professionals, policy makers, and other stakeholders in alleviating pain for children with cancer and their families.

The findings of this review will highlight areas where further research is needed, particularly in developing or validating tools for specific age groups. This can guide future research efforts to fill these gaps.

We will conduct a scoping review following the JBI Manual for Evidence Synthesis to rapidly map the existing evidence related to pain assessment tools for children with cancer. By conducting searches and screening based on rigorous methods, it is possible to comprehensively collect and describe existing evidence. The review will be reported according to PRISMA-ScR, ensuring the provision of necessary information. However, potential limitations exist. Although a scoping review depends on the quality of the included studies, this study will not assess the risk of bias as the aim of this study is to map existing evidence. Because we plan to conduct a bibliographic database search, it will be challenging to identify textbooks, books, and other publications not included in the databases. Consequently, some relevant literature on pain assessment tools may be missed.

## Appendix

Multimedia Appendix 1 Search strategy for PubMed.

Multimedia Appendix 2 Template tables for data collection.

## Abbreviations

JBI: Joanna Briggs Institute
PCC: Population, Concept, and Context
PRISMA-ScR: Preferred Reporting Items for Systematic Reviews and Meta-Analyses Extension for Scoping Reviews

## Data Availability

All data produced during this study will be included in the comprehensive review article and its supplementary information files.
