# Supplementary material for: Pain Assessment Tools for Infants, Children, and Adolescents With Cancer: Protocol for a Scoping Review
Source: JMIR Res Protoc. 2025 Apr 28;14:e66614. doi: 10.2196/66614 (PMC12070012; doi:10.2196/66614)
Supplement: Multimedia Appendix 1 [file resprot_v14i1e66614_app1.pdf]

Appendix 1. Search strategy for PubMed

(("Cancer Pain"[Mesh] OR "Cancer Associated Pain"[tw] OR "Cancer Associated Pains"[tw] OR "Cancer Pain"[tw] OR "Cancer Pains"[tw] OR "Cancer Related Pain"[tw] OR "Cancer Related Pains"[tw] OR "Neoplasm Associated Pain"[tw] OR "Neoplasm Associated Pains"[tw] OR "Neoplasm Related Pain"[tw] OR "Neoplasm Related Pains"[tw] OR "Oncological Pain"[tw] OR "Oncological Pains"[tw] OR "Oncology Pain"[tw] OR "Oncology Pains"[tw] OR "Tumor Associated Pain"[tw] OR "Tumor Associated Pains"[tw] OR "Tumor Pain"[tw] OR "Tumor Pains"[tw] OR "Tumor Related Pain"[tw] OR "Tumor Related Pains"[tw] OR "Leukemia Pain"[tw] OR "Leukemia Pains"[tw] OR "Leukaemia Pain"[tw] OR "Leukaemia Pains"[tw] OR "Lymphoma Pain"[tw] OR "Lymphoma Pains"[tw] OR "Neuroblastoma Pain"[tw] OR "Neuroblastoma Pains"[tw] OR "Sarcoma Pain"[tw] OR "Sarcoma Pains"[tw] OR "Osteosarcoma Pain"[tw] OR "Osteosarcoma Pains"[tw] OR "Rhabdomyosarcoma Pain"[tw] OR "Rhabdomyosarcoma Pains"[tw] OR "Hepatoblastoma Pain"[tw] OR "Hepatoblastoma Pains"[tw] OR "Nephroblastoma Pain"[tw] OR "Nephroblastoma Pains"[tw] OR "Cancer Associated Pain"[title/abstract:~100] OR "Cancer Associated Pains"[title/abstract:~100] OR "Cancer Pain"[title/abstract:~100] OR "Cancer Pains"[title/abstract:~100] OR "Cancer Related Pain"[title/abstract:~100] OR "Cancer Related Pains"[title/abstract:~100] OR "Neoplasm Associated Pain"[title/abstract:~100] OR "Neoplasm Associated Pains"[title/abstract:~100] OR "Neoplasm Related Pain"[title/abstract:~100] OR "Neoplasm Related Pains"[title/abstract:~100] OR "Oncological Pain"[title/abstract:~100] OR "Oncological Pains"[title/abstract:~100] OR "Oncology Pain"[title/abstract:~100] OR "Oncology Pains"[title/abstract:~100] OR "Tumor Associated Pain"[title/abstract:~100] OR "Tumor Associated Pains"[title/abstract:~100] OR "Tumor Pain"[title/abstract:~100] OR "Tumor Pains"[title/abstract:~100] OR "Tumor Related Pain"[title/abstract:~100] OR "Tumor Related Pains"[title/abstract:~100] OR "Leukemia Pain"[title/abstract:~100] OR "Leukemia Pains"[title/abstract:~100] OR "Leukaemia Pain"[title/abstract:~100] OR "Leukaemia Pains"[title/abstract:~100] OR "Lymphoma Pain"[title/abstract:~100] OR "Lymphoma Pains"[title/abstract:~100] OR "Neuroblastoma Pain"[title/abstract:~100] OR "Neuroblastoma Pains"[title/abstract:~100] OR "Sarcoma Pain"[title/abstract:~100] OR "Sarcoma Pains "[title/abstract:~100] OR "Osteosarcoma Pain"[title/abstract:~100] OR "Osteosarcoma Pains "[title/abstract:~100] OR "Rhabdomyosarcoma Pain"[title/abstract:~100] OR "Rhabdomyosarcoma Pains "[title/abstract:~100] OR "Hepatoblastoma Pain"[title/abstract:~100] OR "Hepatoblastoma Pains

"[title/abstract:~100] OR "Nephroblastoma Pain"[title/abstract:~100] OR "Nephroblastoma Pains"[title/abstract:~100]) AND ("Pain Measurement"[Mesh] OR "Self Report"[Mesh] OR "Analgesia Test"[tw] OR "Analgesia Tests"[tw] OR "Analog Pain Scale"[tw] OR "Analog Pain Scales"[tw] OR "Analogue Pain Scale"[tw] OR "Analogue Pain Scales"[tw] OR "assessment instrument"[tw] OR "assessment instruments"[tw] OR "assessment scale"[tw] OR "assessment scales"[tw] OR "assessment tool"[tw] OR "assessment tools"[tw] OR "Formalin Test"[tw] OR "Formalin Tests"[tw] OR "McGill Pain Questionnaire"[tw] OR "McGill Pain Scale"[tw] OR "Nociception Test"[tw] OR "Nociception Tests"[tw] OR "Pain Assessment"[tw] OR "Pain Assessments"[tw] OR "pain evaluation"[tw] OR "Pain Intensities"[tw] OR "Pain Intensity"[tw] OR "Pain Measurement"[tw] OR "Pain Measurements"[tw] OR "Pain Questionnaire"[tw] OR "Pain Questionnaires"[tw] OR "Pain Scale"[tw] OR "Pain Scales"[tw] OR "Pain Severities"[tw] OR "Pain Severity"[tw] OR "Pain Test"[tw] OR "Pain Tests"[tw] OR "parental involvement"[tw] OR "proxy pain assessment"[tw] OR "self report"[tw] OR "self reports"[tw] OR "Tourniquet Pain Test"[tw] OR "Tourniquet Pain Tests"[tw] OR "Visual Analog Pain Scale"[tw] OR "Visual Analogue Pain Scale"[tw] OR "Analgesia Test"[title/abstract:~3] OR "Analgesia Tests"[title/abstract:~3] OR "Analog Pain Scale"[title/abstract:~3] OR "Analog Pain Scales"[title/abstract:~3] OR "Analogue Pain Scale"[title/abstract:~3] OR "Analogue Pain Scales"[title/abstract:~3] OR "assessment instrument"[title/abstract:~3] OR "assessment instruments"[title/abstract:~3] OR "assessment scale"[title/abstract:~3] OR "assessment scales"[title/abstract:~3] OR "assessment tool"[title/abstract:~3] OR "assessment tools"[title/abstract:~3] OR "Formalin Test"[title/abstract:~3] OR "Formalin Tests"[title/abstract:~3] OR "McGill Pain Questionnaire"[title/abstract:~3] OR "McGill Pain Scale"[title/abstract:~3] OR "Nociception Test"[title/abstract:~3] OR "Nociception Tests"[title/abstract:~3] OR "Pain Assessment"[title/abstract:~3] OR "Pain Assessments"[title/abstract:~3] OR "pain evaluation"[title/abstract:~3] OR "Pain Intensities"[title/abstract:~3] OR "Pain Intensity"[title/abstract:~3] OR "Pain Measurement"[title/abstract:~3] OR "Pain Measurements"[title/abstract:~3] OR "Pain Questionnaire"[title/abstract:~3] OR "Pain Questionnaires"[title/abstract:~3] OR "Pain Scale"[title/abstract:~3] OR "Pain Scales"[title/abstract:~3] OR "Pain Severities"[title/abstract:~3] OR "Pain Severity"[title/abstract:~3] OR "Pain Test"[title/abstract:~3] OR "Pain Tests"[title/abstract:~3] OR "parental involvement"[title/abstract:~3] OR "proxy pain assessment"[title/abstract:~3] OR "self report"[title/abstract:~3] OR "self reports"[title/abstract:~3] OR "Tourniquet Pain Test"[title/abstract:~3] OR "Tourniquet Pain Tests"[title/abstract:~3] OR "Visual Analog Pain Scale"[title/abstract:~3] OR "Visual

Analogue Pain Scale"[title/abstract:~3] OR "Parents"[mesh] OR "parent"[tw] OR "parents"[tw] OR "parental"[tw] OR "mother"[tw] OR "mothers"[tw] OR "father"[tw] OR "fathers"[tw]) AND ("Child"[Mesh] OR "child"[tw] OR "children"[tw] OR "Infant"[Mesh] OR "infant"[tw] OR "infants"[tw] OR "infancy"[tw] OR "newborn"[tw] OR "newborns"[tw] OR "new-born"[tw] OR "new-borns"[tw] OR "neonate"[tw] OR "neonates"[tw] OR "neonatal"[tw] OR "neo-nate"[tw] OR "neo-nates"[tw] OR "neo-natal"[tw] OR "neonatology"[tw] OR "NICU"[ti] OR "premature"[tw] OR "prematures"[tw] OR "pre-mature"[tw] OR "pre-matures"[tw] OR "preterm"[tw] OR "pre-term"[tw] OR "postnatal"[tw] OR "post-natal"[tw] OR "baby"[tw] OR "babies"[tw] OR "suckling"[tw] OR "sucklings"[tw] OR "toddler"[tw] OR "toddlers"[tw] OR "childhood"[tw] OR "schoolchild"[tw] OR "schoolchildren"[tw] OR "childcare"[tw] OR "child-care"[tw] OR "young"[ti] OR "youngster"[tw] OR "youngsters"[tw] OR "preschool"[tw] OR "pre-school"[tw] OR "kid"[tw] OR "kids"[tw] OR "boy"[tw] OR "boys"[tw] OR "girl"[tw] OR "girls"[tw] OR "Adolescent"[Mesh] OR "adolescent"[tw] OR "adolescents"[tw] OR "adolescence"[tw] OR "pre-adolescent"[tw] OR "pre-adolescents"[tw] OR "pre-adolescence"[tw] OR "schoolage"[tw] OR "schoolboy"[tw] OR "schoolboys"[tw] OR "schoolgirl"[tw] OR "schoolgirls"[tw] OR "pre-puber"[tw] OR "pre-pubers"[tw] OR "pre-puberty"[tw] OR "prepuber"[tw] OR "prepubers"[tw] OR "prepuberty"[tw] OR "puber"[tw] OR "pubers"[tw] OR "puberty"[tw] OR "puberal"[tw] OR "teenager"[tw] OR "teenagers"[tw] OR "teens"[tw] OR "youth"[tw] OR "youths"[tw] OR "underaged"[tw] OR "under-aged"[tw] OR "Pediatrics"[Mesh] OR "Pediatric"[tw] OR "Pediatrics"[tw] OR "Paediatric"[tw] OR "Paediatrics"[tw] OR "PICU"[ti] **OR** ("child"[all fields] NOT child[au]) OR children*[all fields] OR schoolchild*[all fields] OR "infant"[all fields] OR "infants"[all fields] OR "infancy"[all fields] OR adolesc*[all fields] OR pediat*[all fields] OR paediat*[all fields] OR neonat*[all fields] OR toddler*[all fields] OR "teen"[all fields] OR "teens"[all fields] OR teenager*[all fields] OR preteen*[all fields] OR newborn*[all fields] OR postneonat*[all fields] OR postnatal*[all fields] OR "puberty"[all fields] OR preschool*[all fields] OR suckling*[all fields] OR "juvenile"[all fields] OR "new born"[all fields] OR "new borns"[all fields] OR new-born*[all fields] OR neo-nat*[all fields] OR neonat*[all fields] OR perinat*[all fields] OR underag*[all fields] OR "under age"[all fields] OR "under aged"[all fields] OR youth*[all fields] OR kinder*[all fields] OR pubescen*[all fields] OR prepubescen*[all fields] OR "prepuberty"[all fields] OR "school age"[all fields] OR "schoolage"[all fields] OR "school ages"[all fields] OR schoolage*[all fields] OR "one year old"[ti] OR "two year old"[ti] OR "three year old"[ti] OR "four year old"[ti] OR "five year old"[ti] OR "six year old"[ti] OR "seven year old"[ti] OR "eight year old"[ti] OR "nine year old"[ti] OR "ten year old"[ti]

OR "eleven year old"[ti] OR "twelve year old"[ti] OR "thirteen year old"[ti] OR "fourteen year old"[ti] OR "fifteen year old"[ti] OR "sixteen year old"[ti] OR "seventeen year old"[ti] OR "eighteen year old"[ti] OR "1 year old"[ti] OR "2 year old"[ti] OR "3 year old"[ti] OR "4 year old"[ti] OR "5 year old"[ti] OR "6 year old"[ti] OR "7 year old"[ti] OR "8 year old"[ti] OR "9 year old"[ti] OR "10 year old"[ti] OR "11 year old"[ti] OR "12 year old"[ti] OR "13 year old"[ti] OR "14 year old"[ti] OR "15 year old"[ti] OR "16 year old"[ti] OR "17 year old"[ti] OR "18 year old"[ti] OR "two years old"[ti] OR "three years old"[ti] OR "four years old"[ti] OR "five years old"[ti] OR "six years old"[ti] OR "seven years old"[ti] OR "eight years old"[ti] OR "nine years old"[ti] OR "ten years old"[ti] OR "eleven years old"[ti] OR "twelve years old"[ti] OR "thirteen years old"[ti] OR "fourteen years old"[ti] OR "fifteen years old"[ti] OR "sixteen years old"[ti] OR "seventeen years old"[ti] OR "eighteen years old"[ti] OR "2 years old"[ti] OR "3 years old"[ti] OR "4 years old"[ti] OR "5 years old"[ti] OR "6 years old"[ti] OR "7 years old"[ti] OR "8 years old"[ti] OR "9 years old"[ti] OR "10 years old"[ti] OR "11 years old"[ti] OR "12 years old"[ti] OR "13 years old"[ti] OR "14 years old"[ti] OR "15 years old"[ti] OR "16 years old"[ti] OR "17 years old"[ti] OR "18 years old"[ti]))
