# Supplementary material for: Pain Assessment Tools for Infants, Children, and Adolescents With Cancer: Protocol for a Scoping Review
Source: JMIR Res Protoc. 2025 Apr 28;14:e66614. doi: 10.2196/66614 (PMC12070012; doi:10.2196/66614)
Supplement: Multimedia Appendix 2 [file resprot_v14i1e66614_app2.docx]

Table S1. Dummy Table of Characteristics of Included Studies

| Study ID | Author, publication year | Title | Purpose of studies | Study setting | Study population | Assessment tools | Study design | Findings |
| --- | --- | --- | --- | --- | --- | --- | --- | --- |
| 1 |  |  |  |  |  |  |  |  |
| 2 |  |  |  |  |  |  |  |  |
| 3 |  |  |  |  |  |  |  |  |

Table S2. Dummy Table of Characteristics of Assessment Tools

| Study ID | Author, publication year | Types of tools | Target population | Assessors | Validity and reliability | Instruction | Precautions | Advantages and disadvantages |
| --- | --- | --- | --- | --- | --- | --- | --- | --- |
| 1 |  |  |  |  |  |  |  |  |
| 2 |  |  |  |  |  |  |  |  |
| 3 |  |  |  |  |  |  |  |  |
